# Production of Proteolytic Enzymes by a Keratin-Degrading *Aspergillus niger*

**DOI:** 10.4061/2011/487093

**Published:** 2011-10-10

**Authors:** Fernanda Cortez Lopes, Lucas André Dedavid e Silva, Deise Michele Tichota, Daniel Joner Daroit, Renata Voltolini Velho, Jamile Queiroz Pereira, Ana Paula Folmer Corrêa, Adriano Brandelli

**Affiliations:** ^1^Laboratório de Bioquímica e Microbiologia Aplicada, Departamento de Ciência de Alimentos (ICTA), Universidade Federal do Rio Grande do Sul, Avenida Bento Gonçalves 9500, 91501-970 Porto Alegre, RS, Brazil; ^2^Laboratório de Peptídeos e Enzimas Proteolíticas, Centro de Biotecnologia, Universidade Federal do Rio Grande do Sul, Avenida Bento Gonçalves 9500, 91501-970 Porto Alegre, RS, Brazil

## Abstract

A fungal isolate with capability to grow in keratinous substrate as only source of carbon and nitrogen was identified as *Aspergillus niger* using the sequencing of the ITS region of the rDNA. This strain produced a slightly acid keratinase and an acid protease during cultivation in feather meal. The peak of keratinolytic activity occurred in 48 h and the maximum proteolytic activity in 96 h. These enzymes were partly characterized as serine protease and aspartic protease, respectively. The effects of feather meal concentration and initial pH on enzyme production were evaluated using a central composite design combined with response surface methodology. The optimal conditions were determined as pH 5.0 for protease and 7.8 for keratinase and 20 g/L of feather meal, showing that both models were predictive. Production of keratinases by *A. niger* is a less-exploited field that might represent a novel and promising biotechnological application for this microorganism.

## 1. Introduction

*Aspergillus niger* is one of the most important microorganisms in biotechnology. It has been already used to produce extracellular enzymes such as glucose oxidase, pectinase, *α*-amylase and glucoamylase, organic acids, and recombinant proteins. In addition, *A. niger* is used for biotransformations and waste treatment [1–3]. Among the various enzymes produced by the fungus are included proteases. The major extracellular proteolytic activities in *A. niger* appear to be due to acid proteases [4]. Acid proteases [E.C.3.4.23] are endopeptidases that depend on aspartic acid residues for their catalytic activity and show maximal activity at low pH. These enzymes offer a variety of applications in the food, beverage industry, and medicine [5].

Keratin is a fibrous protein that occurs in vertebrates and exerts protective and structural functions. It is the major component of feathers, wool, scales, hair, *stratum corneum*, horns, scalps, and nails [6]. Keratin is insoluble and presents high mechanic resistance, as well as recalcitrance to common proteolytic enzymes like pepsin, trypsin, and papain [7]. This resistance is because of the tight folding of protein chain in *α*-helix (*α*-keratin) and *β*-sheets (*β*-keratin) in a super-coiled polypeptide chain, kept by strong association by disulfide bonds [8, 9]. Keratinases [EC 3.4.21/24/99.11] are specific proteases that display the capability of keratin degradation. These enzymes are gaining importance in the last years, with many applications associated with hydrolysis of keratinous substrates, mainly byproducts of agroindustrial processes [10]. Generally, keratinases have optimum pH from neutral to alkaline [11].

The utilization of agroindustrial wastes may represent an added value to the industry and meets the increasing awareness for energy conserving and recycling [12]. This fact stimulates the investigation for alternatives to convert keratinous waste into valuable products [10]. One example is the poultry industry that generates huge amount of byproducts, which may represent a potential environmental hazard if they are incorrectly destined or processed. The processing and/or treatment of slaughterhouse waste have been one of the great concerns of poultry industry, mainly because of the restrictions on environmental questions [13].

In this work, the production of proteolytic enzymes by a new keratinolytic strain of *A. niger* was investigated. The enzyme activity was partially characterized, and a culture medium based on keratinous substrate was selected, evaluating the influence of growth substrate concentration and medium pH on the production of proteolytic enzymes.

## 2. Materials and Methods

### 2.1. Microorganism

The fungus used in this work was isolated from citrus waste and belongs to the collection of Laboratório de Bioquímica e Microbiologia Aplicada (Porto Alegre, Brazil). The strain was maintained on Potato Dextrose Agar (PDA; Himedia, Mumbai, India) slants with mineral oil at 4°C and subcultured periodically. 

### 2.2. DNA Isolation

Genomic DNA was extracted according to the method described by Casali et al. [14] with modifications. A culture of 5 days in 5 mL of brain heart infusion broth (BHI; Oxoid, Hampshire, England) was vortexed, and the whole mycelia was transferred to a microtube containing 0.5 mL of TENTS (2% Triton X-100; 1 mM EDTA; 200 mM NaCl; 10 mM Tris-HCl Buffer pH 7.5; 1% SDS) and 0.2 mL of glass beads (200 *μ*m). This mixture was vortexed for 4 cycles of 2 min alternating with ice bath of 30 s. After 0.5 mL of chloroform was added, it was vortexed for 30 s and centrifuged for 15 min at 13,000 × g. The aqueous phase was washed again with chloroform. The nucleic acids were precipitated with 2 vol of absolute ethanol containing 200 mM NaCl. The microtubes were incubated at −20°C overnight and centrifuged for 15 min at 13,000 × g. The precipitate was washed with 0.2 mL of 70% (v/v) ethanol. After ethanol evaporation, the precipitate was resuspended in 20 *μ*L MilliQ water. The extracted DNA was observed on 0.9% agarose gels. 

### 2.3. PCR Assay

The fungus was identified using the partial sequencing of the intergenic region of the rDNA. An ITS region was selected as a target sequence for PCR using universal primers *ITS 1* (5′-TCCGTAGGTGAACCTGCGG-3′) as a forward primer and *ITS 4* (5′-TCCTCCGCTTTATTGATATGC-3′) as a reverse primer. A PCR using the thermocycler (Eppendorf, Hamburg, Germany) was performed with the following parameters: an initial denaturation of 5 min at 95°C, 30 cycles of 30 s at 95°C for denaturation, 30 s at 46°C for annealing, 80 s at 72°C for extension, and a final extension of 7 min at 72°C, according to Horisawa et al. [15] with modifications. All PCR products were stored at −20°C until analyzed. Aliquots of PCR products were examined after electrophoresis on 0.9% agarose gels.

### 2.4. DNA Sequencing

PCR products were sequenced in the ACTGene Laboratory (*Centro de Biotecnologia*, UFRGS, Porto Alegre, Brazil) using the automatic sequencer ABI-PRISM 3100 Genetic Analyzer armed with 50 cm capillaries and POP6 polymer (Applied Biosystems). Sequencing data were collected using the software Data Collection v1.0.1 (Applied Biosystems). The sequences obtained were edited using the software BioEdit (1997–2005 Tom Hall) and then submitted to the BLAST algorithm to retrieve for homologous sequences in GenBank (National Center for Biotechnology Information (http://www.ncbi.nlm.nih.gov/)). 

### 2.5. Phylogenetic Analyses

The sequence data were obtained from Genbank submissions and were used for comparisons with the 599 bp from our isolate. The sequences were aligned using software ClustalX version 2.0 [16], and the phylogenetic analyses were performed using MEGA 4.0 [17] for neighbor-joining and bootstrap analysis. *A. fumigatus* and *A. ellipticus* were used as outgroups. Bootstrap values were generated by 1,000 replications.

### 2.6. Conidia Suspension

*A. niger *was routinely cultivated on PDA plates at 30°C until sporulation. After that, the conidia was suspended with sterile distilled water and collected with a Pasteur's capillary. The material was centrifuged at 10,000 × g for 10 min. The supernatant was discarded, and the spores were suspended again in distilled water. The conidia concentration was determined with a Neubauer's chamber. The concentration used in the experiments was 10^6^ conidia/mL [18].

### 2.7. Keratinous Growth Substrates and Culture Media

Five keratinous substrates were used as carbon and nitrogen sources for fungal growth: human hair, pig hair, feather meal, chicken feathers, and bovine horn. Human hair was previously washed with distilled water. Feathers were washed with 0.1% (v/v) Triton X-100 and distilled water and then cut into small pieces to enhance the contact surface. The other substrates were not submitted to pretreatments. 

The keratinous substrates were used in the concentration of 10 g/L in an aqueous solution. The microorganism was cultivated in Erlenmeyer flasks (250 mL) containing 50 mL of medium, for 120 h at 30°C, in a rotary shaker (Marconi, Piracicaba, Brazil) at 120 rpm. Aseptic samples were collected each 24 h and filtered through Whatman filter n*º* 1. The filtrate was used for the enzyme assays.

The medium that provided the higher proteolytic and keratinolytic activity was selected, and then the optimum pH was determined. The buffers used were glycine-HCl (pH 2.5), citrate (pH 3.5–5.5), and phosphate (pH 6.5–8.5), all in the concentration 25 mM. The utilization of this pH 2.5 and 3.5 buffers to protease was not possible because this pH caused azocasein precipitation. The composition analysis of feather meal was developed according to AOAC [19].

### 2.8. Enzymatic Activity on Azocasein

The proteolytic activity was determined using azocasein (Sigma, Steinheim, Germany) as substrate. The assay mixture contained 100 *μ*L of 25 mM citrate buffer pH 4.5, 100 *μ*L of enzyme sample, and 200 *μ*L of 20 mg azocasein/mL in water. A blank tube was prepared with the immediate addition of 800 *μ*L of 20% (w/v) trichloroacetic acid. Then, the tubes were incubated for 15 minutes at 50°C. After incubation, 800 *μ*L of TCA were added to stop the reaction. The tubes were centrifuged for 10 min at 10,000 × g. The activity was estimated spectrophotometrically by reading the absorbance at 400 nm. In this assay, one enzyme unit was expressed as the amount of enzyme that caused an increase of 0.1 in the absorbance at 400 nm per hour [20]. The assays were performed in triplicate.

### 2.9. Enzymatic Activity on Azokeratin

Azokeratin was synthesized as described by Riffel et al. [21]. The assay was conducted for 1 h at 50°C by incubating 100 *μ*L of enzyme sample, and 500 *μ*L of a 20 mg azokeratin/mL suspension in 25 mM citrate buffer, pH 6.5. The reaction was stopped by adding 800 *μ*L of 20% (w/v) TCA solution. The controls were prepared by adding the TCA solution prior to the enzyme. The tubes were centrifuged (10,000 × g for 10 min), and activity was calculated from the increase in absorbance at 440 nm. One enzyme unit was expressed as the amount of enzyme that caused an increase of 0.1 in the absorbance at 440 nm per hour. The assays were performed in triplicate.

### 2.10. Zymography

The samples of 24, 48, 72, 96, and 120 h of cultivation, previously lyophilized and concentrated 10 times, were submitted to electrophoresis on 12% polyacrylamide gels containing gelatin (1 mg/mL). After electrophoresis, gels were washed twice with citrate buffer (25 mM, pH 4.5) or phosphate buffer (25 mM, pH 6.5) containing 2.5% (v/v) Triton X-100 for 30 min, and then with the same buffer without detergent for 45 min. After 12 h of incubation at 30°C, gels were stained with Coomassie Brilliant Blue R-250 during 2 h and then destained. Protease bands appeared as clear zones on a blue background due to gelatin hydrolysis [22].

### 2.11. Characterization Using Protease Inhibitors

Culture supernatants corresponding to peaks of enzymatic activity (48 h for keratinolytic activity and 96 h for proteolytic activity) were characterized using the protease inhibitors EDTA and *o*-phenanthroline (for metalloproteases), PMSF (for serine protease), pepstatin (for aspartic protease), and iodoacetamide (for cysteine protease). Each inhibitor was preincubated with enzyme extracts for 10 min at room temperature before assaying for keratinase or protease activity.

### 2.12. Central Composite Design and Response Surface Methodology

The influence of the keratinous substrate concentration and the initial medium pH on the production of protease and keratinase was evaluated using a 2^2^ experimental model with 4 replicates of the central point, resulting in 12 experiments plus axial points. In the statistical model, the coded variables correspond to feather meal concentration (*X*_1_) and initial pH (*X*_2_), which are the independent variables; the enzymatic activity (*Y*) is the dependent variable, and *b*_i_, *b*_ii_, and *b*_ij_ represent model parameters. The model is demonstrated as follows:

(1) $$\;Y=b_{0}+b_{1}X_{1}+b_{2}X_{2}+b_{12}X_{1}X_{2}+b_{11}X_{1}^{2}+b_{22}X_{2}^{2}.$$

The software Statistica version 6.0 (Statsoft Inc., Tulsa, Okla, USA) was used in the regression analysis of experimental results. The quality of fit of the first-order model equation was expressed by the coefficient of determination *R* ^2^, and its statistical significance was determined by *F*-test (*Fischer's F-Test*).

The production medium (50 mL in 250 mL Erlenmeyer flask) was inoculated as described above and incubated for 48 and 96 h. To adjust the initial pH, media were prepared with the following buffers: 100 mM glycine-HCl (pH 2.2); 100 mM citrate (pH 3.0 and pH 5.0); 100 mM phosphate buffer (pH 7.0 and pH 7.8). 

The existence of a correlation between the predicted and the experimental values justifies the model validity [23]. Initially, substrate concentration and initial pH points were chosen, and for these points, the predicted enzyme activities were calculated using the generated equation. For model validation, these activity values were then compared with enzyme activities obtained in actual experiments.

## 3. Results and Discussion

### 3.1. Identification of the Fungus

**Figure 1:**
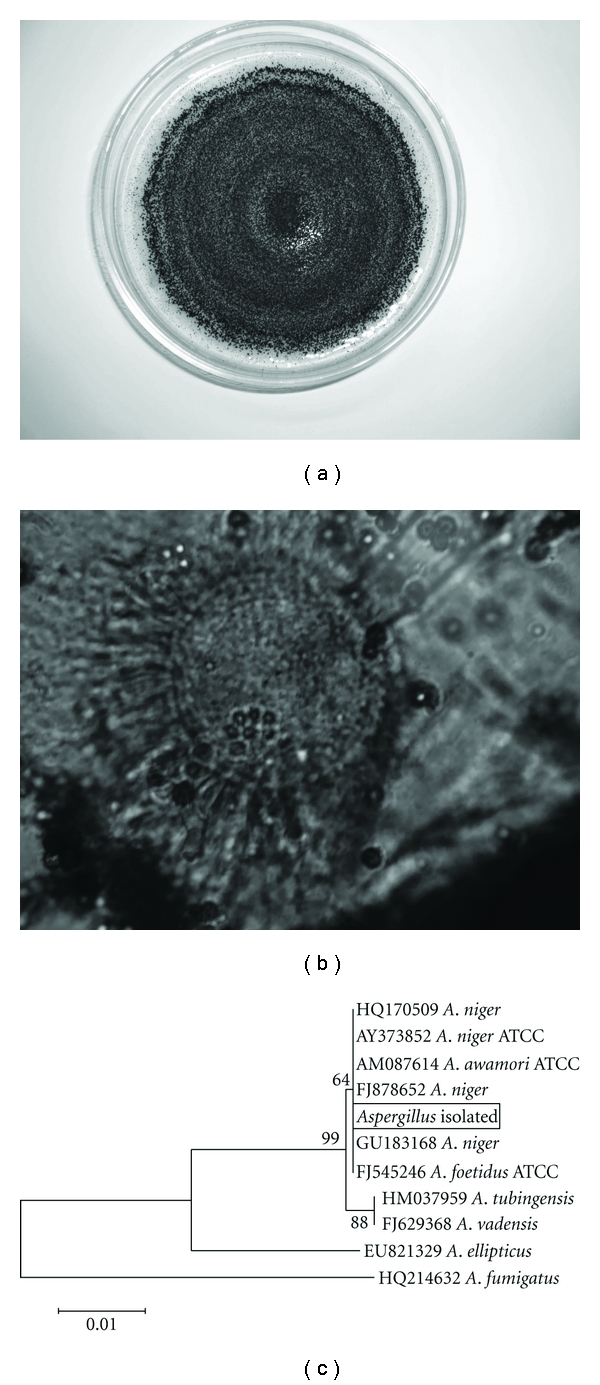
(a) Macromorphology of *A. niger* on PDA. (b) Micromorphology of *A. niger* using optic microscope (1000x). (c) The neighbor-joining tree inferred from the partial sequence of the ITS region data set using Mega 4.0 according to Jukes-Cantor Modell. Bootstrap values from 1000 replications are indicated by the nodes.

The fungus was identified as *Aspergillus niger* using molecular tools. A sequence of 599 bp showed 97% identity with *A. niger. *The phenotypic features are according to the results obtained in the molecular identification, since the micro- and macromorphology (Figures 1(a) and 1(b)) show similarities to those described to *Aspergillus niger* [24]. A phylogenetic tree was developed based on the alignment of the sequences (Figure 1(c)). Giraud et al. [25] reported that *A. awamori* was not a separate species within the *A. niger* aggregate. Besides, the species *A. foetidus*, based on *β*-tubulin analysis, is considered synonyms to *A. niger* [26, 27]. 

According to Schuster et al. [2], the name *A. niger* is predated by the names *Aspergillus phoenicis* and *Aspergillus ficuum*, and it is accepted that these three taxa are all conspecific, as implied by most molecular studies, the latter two taxa would have nomenclatural priority. Since these latter two names are nowadays rarely used, it was proposed at the Second International Workshop on *Penicillium *and *Aspergillus* that the name *A. niger* has to be conserved and *A. phoenicis* and *A. ficuum* have to be rejected. This purpose is justified mainly because of the major economic importance of *A. niger*.

### 3.2. Selection of Culture Medium

**Figure 2:**
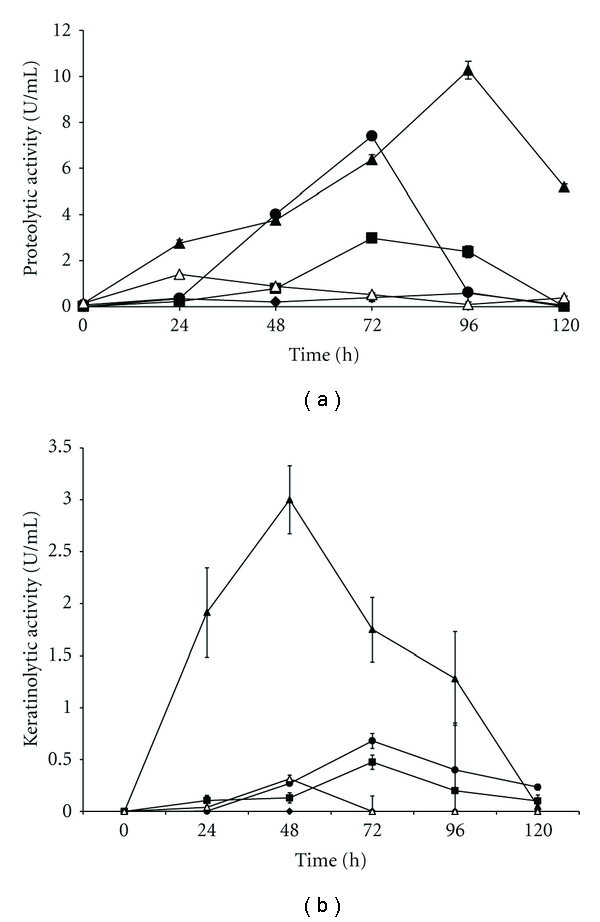
(a) Production of proteolytic activity and (b) production of keratinolytic activity by *A. niger* growing on different keratinous substrates (10 g/L). (■) Bovine horn, (∆) chicken feathers, (▲) feather meal, (*◆*) human hair, and (●) pig hair.

Due to the record of acid protease production by *A. niger*, the production of these proteases during the cultivation (120 h) using different keratinous sources was first investigated. The proteolytic activities observed on different culture media are shown in Figure 2(a). It is possible to observe that the substrate feather meal induced the higher production of proteolytic enzymes (10.27 U/mL) after 96 h of culture. The probable reason could be the higher availability of keratin in feather meal in comparison to the other keratinous materials, mainly because of the thermal and mechanical treatment suffered by this substrate. The process consists in the treatment of feathers, which are pressure cooked in a steam-heated batch cooker at about 207–690 kPa, with a moisture content about 60–70%, for about 30–60 min. This permits the hydrolysis and causes the feathers to break up into a final meal [28]. This product contains a high content of crude protein, ranging between 78 and 92%, being this protein mostly keratin [29]. These values of protein are consistent with the values found in the compositional analysis of the feather meal used in this study (protein 83.7 g/100 g; ash 2.0 g/100 g, fat 4.4 g/100 g).

**Figure 3:**
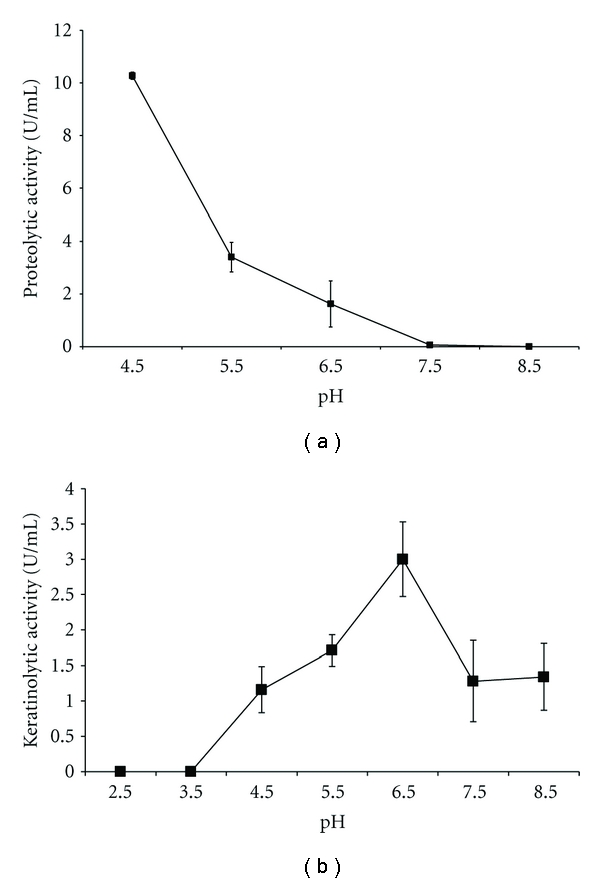
(a) Proteolytic activity of a 96-hour culture and (b) keratinolytic activity of a 48-hour culture of *A. niger* growing on 10 g/L feather meal at different pH (2.5–8.5).

Since *A. niger* was capable of using keratin as the only source of carbon and nitrogen, the production of keratinases was then evaluated (Figure 2(b)). The samples collected at 24, 48, 72, 96, and 120 h were used to determine the peak of keratinolytic activity and the optimum pH. The feather meal substrate also induced the higher keratinolytic activity. The peak of keratinolytic activity occurred in 48 h of cultivation, and the optimum pH was 6.5. The keratinolytic activity in this condition was 3.0 U/mL (Figure 3(b)). Reports on keratinolytic activity of *A. niger* are scarce. The keratinolytic potential of *A. niger* was described by Anbu et al. [30]. Other keratinolytic species belonging to the genus *Aspergillus* were *Aspergillus fumigatus* and *Aspergillus oryzae* [31]. Besides, keratinases generally have optimum pH ranging neutral to alkaline [11, 31]. 

**Figure 4:**
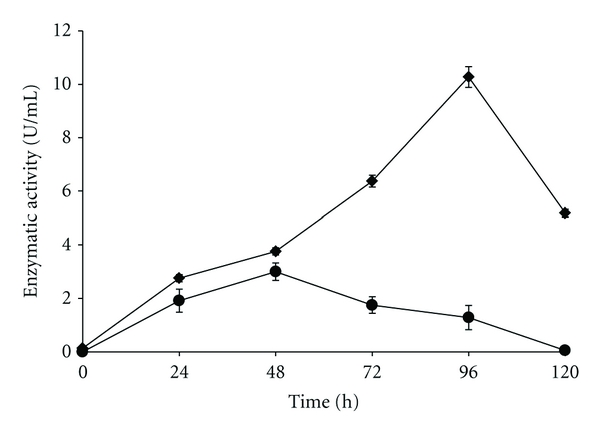
Enzymatic activity of protease (*◆*) and keratinase (●) during the 120-hour culture. The assay was performed in the optimum pH of each enzyme.

From the different cultivation times required for maximal production of keratinase (48 h) and protease (96 h) by *A. niger *(Figure 4), a possible mechanism of feather meal utilization by this strain could be suggested. The probable mechanism of the feather meal degradation could be described as the synergistic activity of both keratinase, which hydrolyzes keratin, a more refractory protein to render polypeptides of less complexity that could be substrate for other protease(s). Moreover, the maximal activity of these enzymes over the time interval analyzed in this study suggests that keratinase would be the first to act, followed by other proteolytic enzymes.

### 3.3. Partial Characterization of Crude Enzymes

To characterize the proteolytic enzymes present in the crude extract, protease inhibitors were used to assay the samples obtained at 48 and 96 h. The proteolytic activity was only inhibited in the presence of pepstatin (Table 1), suggesting an aspartic protease. This is in agreement with the optimum pH of 4.5 (Figure 3(a)), since acidic pH values are characteristic of aspartic proteases. Siala et al. [32] reported the purification of an aspartic protease produced by *A. niger* I1 with optimum pH 3.0, and another study described the production of acid proteases along the growth phases of *A. niger* NRRL 1785 in a five-day cultivation period. The fungus produced a maximum proteolytic activity in 96 h of culture, and the protease was characterized as an aspartic protease with optimum pH 4.0 [33]. These results are in good agreement with those found in the present study. 

Regarding the keratinolytic activity, the inhibition was observed using PMSF, resulting in a residual activity of 58%. Most of the microbial keratinases are alkaline or neutral proteases showing optima pH ranging from 7.5 to 9.0. However, some enzymes are optimally active outside this range, even at extreme alkalophilic pH or at slightly acidic pH [11, 31]. Keratinases mostly belong to the class of serine or metalloproteases irrespective of the microorganism. Therefore, PMSF, EDTA, and 1,10-phenantroline are potential inhibitors of these enzymes [11]. In this study, the keratinolytic enzyme showed a neutral pH optimum (pH 6.5) and a serine protease character. The keratinases of the genus *Aspergillus* reported in the literature are a serine protease with optimal pH varying between 6.5 and 9.0 for *A. fumigatus* [34] and a metalloprotease produced by *A. oryzae* with optimum pH 8.0 [35].

**Figure 5:**
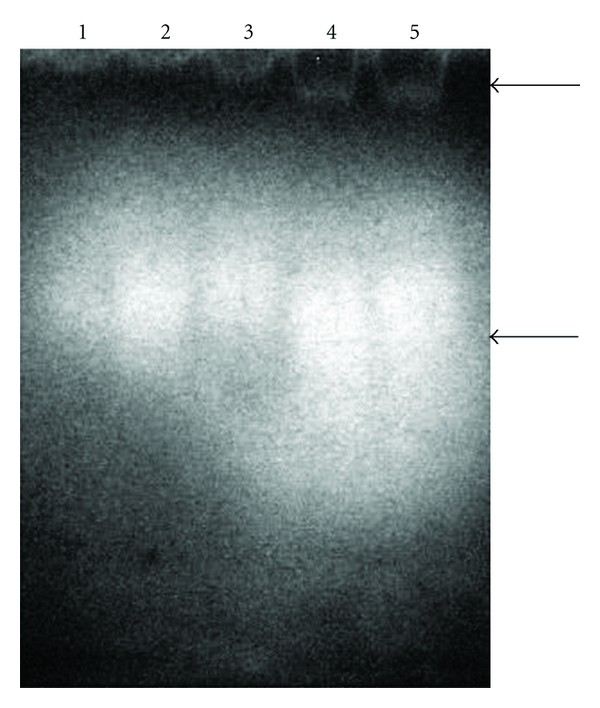
Zymogram analysis of (1) 24, (2) 48, (3) 72, (4) 96, and (5) 120-hour cultures supernatants of *A. niger* using citrate buffer pH 4.5.

The zymogram revealed two bands, indicating the presence of two proteases (Figure 5). The same activity pattern was observed in the gels incubated in different buffers (data not shown). 

### 3.4. Response Surface Methodology

The experimental conditions and the results of RSM are presented in Table 2. In this study, setting the keratinous substrate (feather meal) and initial pH as variables, the higher keratinolytic activity was 1.73 U/mL with 20 g/L feather meal and pH 7.8, and the proteolytic activity was 12.03 U/mL using 20 g/L of feather meal and pH 5.0. 

Using the software Statistica 6.0, it was possible to analyze the effect of the independent variables on enzyme production with confidence level of 90% and *R^2^* of 0.90 for protease and 95% and *R^2^* of 0.93 for keratinase (Tables 3 and 4, resp.). Deviation from the central points (20 g feather meal/L and pH 5.0) usually resulted in less protease production. The linear effect of feather meal concentration was the most expressive variable for protease production, increasing the enzymatic activity in 4.19 U/mL (Table 3), but was not significant for the keratinase production (Table 4). The axial feather meal points were shown to negatively affect enzyme yields. Although acidic initial pH values were demonstrated to enhance protease production when compared to near-neutrality pH values (Table 3), the opposite was observed for keratinase production, that is, the yield of keratinase tended to be higher at near-neutrality pH (pH 7.0–7.8; Table 4). In fact, the initial pH was the most important variable for keratinase production. The interaction between pH and feather meal concentration was not significant for both activities.

**Figure 6:**
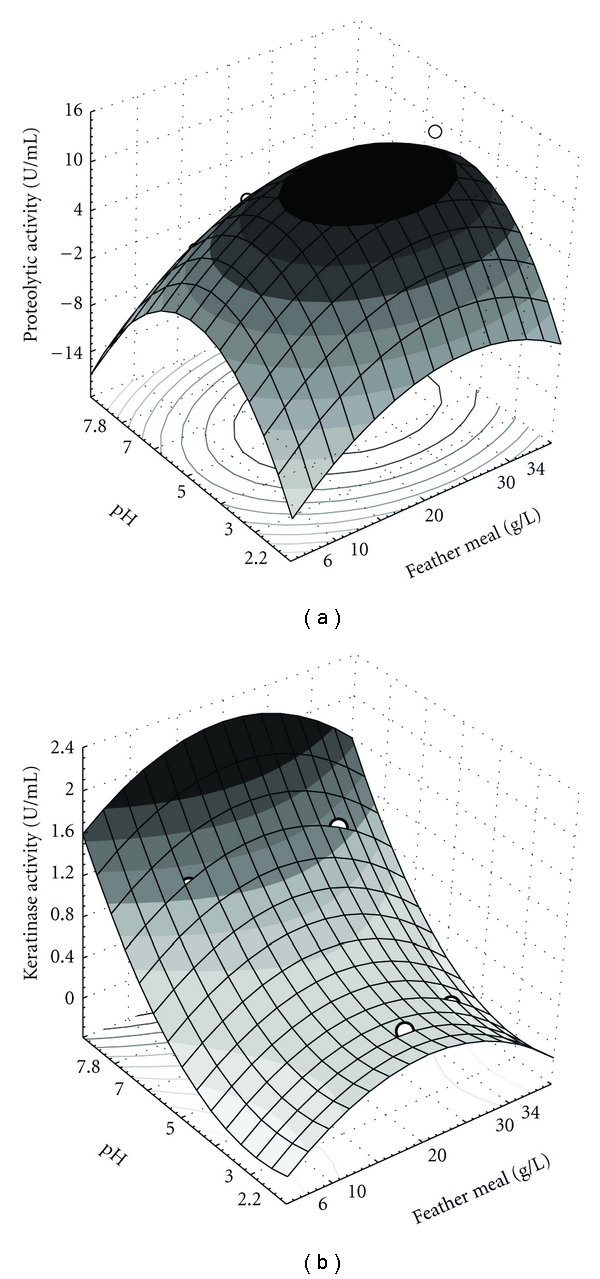
Response surface plots of protease (a) and keratinase (b) production by *A. niger* as a function of pH and feather meal concentration.

To construct a second-order model, which can predict the enzymatic activity as a function of the independent variables, the variance analysis (ANOVA) was used to evaluate the model significance. The calculated* F*-value was 33.05 for keratinase and 18.13 for protease, whereas the tabulated *F*-value was 4.76 and 3.18, respectively. As the calculated *F*-values were higher approximately 7 and 6 times than the tabulated *F*-value, the model is predictive. For biological assays, the calculated *F*-value must be higher than 3 times of tabulated *F*-value; thus, the model is considered predictive and significant [36]. It was possible to generate the surface graphics (Figure 6) through (2) and (3), describing the keratinolytic and proteolytic activities as a function of the independent variables

(2) $$\begin{array}{c} Y_{\text{keratinase}}=0.70-0.18x_{1}^{2}+0.48x_{2}+0.19x_{2}^{2}, \end{array}$$

(3) $$\begin{array}{c} Y_{\text{protease}}=11.02+2.10x_{1}-2.27x_{1}^{2}-0.70x_{2}-4.84x_{2}^{2}. \end{array}$$

The values of the keratinase activity obtained in the factorial design were smaller than the cultures without initial pH adjust. The conditions chosen in the experimental design were more favorable to protease production, obtaining an increase in the proteolytic activity. Nevertheless, the equations obtained in this study could predict the enzymatic activity of each enzyme and could be used to optimize conditions of the bioprocess.

To validate the RSM, four points were chosen, and experimental and predicted enzymatic activities were determined to these points (Table 5). In biological processes, a difference until 20% between experimental and predicted results is acceptable [37], and as all values are within this range, the model describes adequately the influence of feather meal concentration and pH on enzyme production. 

Feather meal was selected as substrate for protease and keratinase production by *A. niger.* This strain produces an aspartic protease with general proteolytic activity and a serine protease with keratinolytic activity. The optima conditions were different for the production of each enzyme, and the models were validated and can predict the enzymatic activity. *A. niger* has potential to be used in biotechnological process involving keratin hydrolysis, mainly because of the GRAS status of these products. Thus, more studies are necessary to better exploit this keratinolytic potential.

## Figures and Tables

**Table 1:** Characterization of proteolytic and keratinolytic activity using protease inhibitors.

		Residual activity (%)	
Inhibitor	Concentration	Proteolytic	Keratinolytic
None	—	100	100
EDTA	5 mM	83	176
PMSF	5 mM	99	58
1,10-phenantroline	1 mM	107	145
Pepstatin	5 *μ*M	18	100
Iodoacetamide	100 *μ*M	89	92

**Table 2:** Experimental design and results of the 2^2^ factorial design.

Run	*X*_1_ (concentration, g/L)	*X*_2_ (pH)	*Y*_1_ (proteolytic activity U/mL)	*Y*_2_ (keratinolytic activity U/mL)
1	−1 (10)	−1 (3)	2.25	0
2	−1 (10)	+1 (7)	0.38	1.19
3	+1 (30)	−1 (3)	3.69	0.29
4	+1 (30)	+1 (7)	4.32	1.22
5	0 (20)	0 (5)	10.87	0.54
6	0 (20)	0 (5)	10.36	0.84
7	0 (20)	0 (5)	12.03	0.75
8	0 (20)	0 (5)	10.80	0.68
9	0 (20)	−1.41 (2.20)	4.17	0.52
10	0 (20)	+ 1.41 (7.80)	1.12	1.73
11	−1.41 (6)	0 (5)	3.73	0.55
12	+1.41 (34)	0 (5)	11.77	0.20

**Table 3:** Main effects and interaction analysis for protease production by *A. niger* growing on feather meal.

Factors	Effect protease	Standard error	*t *value	*P* value
Medium	11.02078	0.355983	30.9588	0.000074*
Feather meal (*L*)	4.19157	0.504191	8.3135	0.003647*
Feather meal (*Q*)	−4.54622	0.565214	−8.0434	0.004013*
pH (*L*)	−1.39211	0.504191	−2.7611	0.070089*
pH (*Q*)	−9.68345	0.565214	−17.1324	0.000433*
Feather meal × pH	1.25000	0.711972	1.7557	0.177405

*R* ^2^ = 0.90.

*Significant factors, *P* < 0.1.

**Table 4:** Main effects and interaction analysis for keratinase production by *A. niger* growing on feather meal.

Factors	Effect keratinase	Standard error	*t *value	*P* value
Medium	0.702720	0.063294	11.10241	0.001566*
Feather meal (*L*)	−0.045272	0.089646	−0.50501	0.648314
Feather meal (*Q*)	−0.369430	0.100496	−3.67606	0.034853*
pH (*L*)	0.959379	0.089646	10.70186	0.001744*
pH (*Q*)	0.387574	0.100496	3.85660	0.030803*
Feather meal × pH	−0.130000	0.126590	−1.02694	0.380013

*R* ^2^ = 0.93.

*Significant factors, *P* < 0.05.

**Table 5:** Experiments for model validation.*

			Enzymatic activity		
Run**	Feather meal (g/L)	pH	Experimental	Predicted	Difference (%)
1	26	6.2	1.01	0.99	1.99
			9.03	10.94	17.46
2	30	6.2	0.97	0.88	9.28
			7.92	8.69	8.86
3	26	5.0	0.55	0.63	13.00
			11.04	11.46	3.66
4	26	9.0	2.59	2.35	10.21
			0	−9.30	0

*Models for keratinase and protease production are represented by (2) and (3), respectively (see text).

**The first line for each run shows the keratinolytic activity and the second line the proteolytic activity.
